# Supplementary material for: Mendelian Randomization Analyses of Chronic Immune-Mediated Diseases, Circulating Inflammatory Biomarkers, and Cytokines in Relation to Liver Cancer
Source: Cancers (Basel). 2023 May 26;15(11):2930. doi: 10.3390/cancers15112930 (PMC10251825; doi:10.3390/cancers15112930)
Supplement: Supplementary file 1 [file cancers-15-02930-s001.zip › cancers-2399438-supplementary.pdf]

<u>Supplementary materials</u>

Mendelian Randomization Analyses of Chronic Immune-Mediated Diseases, Circulating Inflammatory Biomarkers, and Cytokines in Relation to Liver Cancer

Table of content

Table S1. GWAS information for eight immune-mediated diseases. 2
Table S2. GWAS information for circulating inflammatory biomarkers. 5
Table S3. Associations of circulating inflammatory cytokines with liver cancer according to Mendelian randomization analysis. 6
Table S4. Associations of inflammatory traits with liver cancer (UKBB) according to Mendelian randomization analysis. 13

Table S1. GWAS information for eight immune-mediated diseases.

Chronic immune-mediated disease	GWAS information							
	PMID	Case number	Sample size	SNPs passing QC	Web source*	Source of study population	Case definitions	GWAS analysis
Asthma	32296059	64,538	303,859	9,572,556	GCST010042	The UK Biobank cohort	Doctor diagnosed asthma, International Classification of Diseases version-10 (ICD10) J45 (asthma)/J46 (severe asthma), and self-reported asthma	A GWAS analysis was performed with BOLT-LMM v2.3.2 using a standard (infinitesimal) mixed model to correct for structure due to relatedness, ancestral heterogeneity, with adjustment for age, sex, the first 20 principal components, and genotyping array
Rheumatoid arthritis	33310728	14,361	58,284	13,108,512	GCST90013534	18 studies of Europeans	NR	Associations of SNPs with rheumatoid arthritis were evaluated by logistic regression models assuming additive effects of the allele dosages including top 5 or 10 principal components as covariates (if available) using mach2dat v.1.0.16
Type 1 diabetes	34012112	18,942	520,580	62,116,689	GCST90014023	19 studies of Europeans	NR	The authors tested variants with MAF > 1e−5 for association to T1D with Firth bias reduced logistic regression using EPACTS (https://genome.sph.umich.edu/wiki/EPACTS) for non-UK Biobank cohorts or SAIGE (version 0.38) for the UK Biobank, using genotype dosages adjusted for sex and the first four ancestry PCs.
Psoriasis	23143594	10,588	33,394	111,236	GCST005527	5 studies of Europeans	NR	The GWAS data sets underwent quality control as previously described and were analyzed for association using the top principal components from the previous analyses as covariates
Crohn's disease	28067908	12,194	40,266	9,560,910	GCST004132	a UK cohort	diagnosed using accepted endoscopic, histopathological and radiological criteria	The authors tested for association with ulcerative colitis, Crohn's disease and IBD separately within the sequenced samples and new GWAS using

								SNPTEST v2.5, performing an additive frequentist association test conditioned on the first ten principal components for each cohort.
Ulcerative colitis	28067908	12,366	45,975	9,578,670	GCST004133	a UK cohort	diagnosed using accepted endoscopic, histopathological and radiological criteria	The authors tested for association with ulcerative colitis, Crohn's disease and IBD separately within the sequenced samples and new GWAS using SNPTEST v2.5, performing an additive frequentist association test conditioned on the first ten principal components for each cohort.
Celiac disease	20190752	4,533	15,283	292,387	GCST000612	an European cohort	Affected celiac individuals were diagnosed according to standard clinical, serological and histopathological criteria, including small intestinal biopsy	Analyses were performed using PLINK v1.07, mostly using the Cochran-Mantel-Haenzel test.
Multiple sclerosis	24076602	14,198	38,582	161,311	GCST005531	International Multiple Sclerosis Genetics Consortium (IMSGC)	NR	The authors applied logistic regression, assuming a per-allelic genetic model for each data set, including the first five principal components as covariates to correct for population stratification, and then performed an inverse variance meta-analysis of the 11 strata under a fixed-effects model, as implemented in PLINK
Systemic lupus erythematosus	26502338	5,201	14,267	644,674	GCST003156	From southern Europe, matching the Spanish, Italian and Turkish cases with controls from the same countries.	Diagnosed on the basis of standard American College of Rheumatology (ACR) classification criteria	All case-control analysis was carried out using the SNPTEST algorithm; we used a standard threshold of $P = 5 \times 10{-}8$ for reporting genome-wide significance throughout. The inverse variance method was used for meta-analysis.

Periodontitis	31235808	17,353	45,563	>8,900,000	GCST008300	17,353 European and Hispanic/Latino ancestry cases, 28,210 European and Hispanic/Latino ancestry controls in the GLIDE consortium	For periodontal status, participants were classified as having (cases) or not having (reference) clinical symptoms of periodontitis, using definitions applied by each participating cohort.	For the binary trait of periodontitis, age, age-squared and other study-specific covariates were instead included as covariates in association tests. Every genotyped and imputed SNP was tested for association with these transformed variables using linear regression and additive genetic models, implemented via several software tools

*All GWAS summary data were downloaded from GWAS Catalog. NR, not report.

Table S2. GWAS information for circulating inflammatory biomarkers.

Biomarker name	PMID	Sample size	SNPs passing QC	Study populations	GWAS information	Web source
C-reactive protein	33462484	362,443	801,7993	The UK Biobank cohort	The authors employed a GWAS with covariates of the population-specific PCs and the genotyping array on the residuals computed above. Variants were the full set of HRC-imputed SNPs in the version 3 UK Biobank data release.	Footnotes1
Eosinophil count	27863252	172,275	~ 2,500,000	The UK Biobank and INTERVAL studies	The authors performed a univariable GWAS for each of the 36 blood cell indices that had phenotype data measured or derived in all three studies. Specifically, we computed the association statistics (i.e an estimate of the regression coefficient and the corresponding standard error) for a mixed linear regression of phenotype on the probabilistic imputed allele dose (i.e., an additive model) separately for each of the three datasets using BOLT-LMM v2.2 (Loh et al., 2015). The linear mixed model accounts for the genetic component of phenotypic correlation generated by relatedness. In order to maximize protection against confounding by large scale relatedness, we included a dummy variable for each recruitment center and the first ten PCs of the study specific kinship matrices as covariates in each regression model.	GCST004606
Leukocyte count		172,435				GCST004610
Basophil count		171,846				GCST004618
Lymphocyte count		171,643				GCST004627
Monocyte count		170,721				GCST004625
Neutrophil count		170,702				GCST004629

Footnotes1: https://nih.figshare.com/articles/dataset/The_meta-analyzed_GWAS_summary_statistics_for_35_lab_biomarkers_described_in_Genetics_of_35_blood_and_urine_biomarkers_in_the_UK_Biobank_/12355382

Table S3. Associations of circulating inflammatory cytokines with liver cancer according to Mendelian randomization analysis.

ID*	Protein	method	No. of IVs	Biomarker	Full name	Group	OR (95% CI)	P value
11593_21	CXCL9	IVW	4	MIG	C-X-C motif chemokine 9	Chemokine	1.37 (0.47 to 3.95)	0.5622
13748_4	CCL8	IVW	81	MCP-2	C-C motif chemokine 8	Chemokine	0.90 (0.78 to 1.04)	0.1611
18289_16	CCL15	IVW	54	MIP-5	C-C motif chemokine 15	Chemokine	0.98 (0.84 to 1.14)	0.7543
2192_63	CCL27	IVW	2	CTACK	C-C motif chemokine 27	Chemokine	1.55 (0.26 to 9.12)	0.6294
**2436_49**	**CXCL16**	**IVW**	**41**	**CXCL16, soluble**	**C-X-C motif chemokine 16**	**Chemokine**	**0.78 (0.61 to 1.00)**	**0.0463**
2516_57	CCL21	IVW	56	6Ckine	C-C motif chemokine 21	Chemokine	1.00 (0.83 to 1.21)	0.9727
2578_67	CCL2	IVW	4	MCP-1	C-C motif chemokine 2	Chemokine	0.41 (0.17 to 1.04)	0.0594
2705_5	CCL25	IVW	56	TECK	C-C motif chemokine 25	Chemokine	0.95 (0.81 to 1.11)	0.5255
2770_51	CCL1	IVW	22	I-309	C-C motif chemokine 1	Chemokine	0.97 (0.67 to 1.40)	0.8647
2781_63	CCL4L1	IVW	11	LAG-1	C-C motif chemokine 4-like	Chemokine	1.21 (0.77 to 1.90)	0.3989
2900_53	CCL14	IVW	30	HCC-1	C-C motif chemokine 14	Chemokine	1.00 (0.80 to 1.24)	0.9745
2913_1	CCL23	IVW	25	MPIF-1	C-C motif chemokine 23	Chemokine	0.98 (0.81 to 1.18)	0.7988
2979_8	CXCL5	IVW	8	ENA-78	C-X-C motif chemokine 5	Chemokine	1.00 (0.58 to 1.73)	0.9966
3038_9	CXCL11	IVW	36	I-TAC	C-X-C motif chemokine 11	Chemokine	0.94 (0.73 to 1.22)	0.6431
3040_59	CCL3	IVW	11	MIP-1a	C-C motif chemokine 3	Chemokine	1.07 (0.63 to 1.82)	0.8101
3044_3	CCL18	IVW	48	PARC	C-C motif chemokine 18	Chemokine	0.99 (0.84 to 1.17)	0.9046
3487_32	CXCL13	IVW	4	BLC	C-X-C motif chemokine 13	Chemokine	1.54 (0.34 to 7.00)	0.5774
3495_15	CXCL6	IVW	70	GCP-2	C-X-C motif chemokine 6	Chemokine	0.95 (0.82 to 1.11)	0.5343
3508_78	CCL22	IVW	33	MDC	C-C motif chemokine 22	Chemokine	1.09 (0.79 to 1.49)	0.6095
3519_3	CCL17	IVW	44	TARC	C-C motif chemokine 17	Chemokine	1.01 (0.83 to 1.24)	0.9014
4141_79	CXCL10	IVW	11	IP-10	C-X-C motif chemokine 10	Chemokine	0.89 (0.54 to 1.47)	0.6508
4144_13	CCL13	IVW	2	MCP-4	C-C motif chemokine 13	Chemokine	1.15 (0.29 to 4.62)	0.8433
4886_3	CCL7	IVW	42	MCP-3	C-C motif chemokine 7	Chemokine	1.13 (0.94 to 1.37)	0.2007
4913_78	CCL16	IVW	64	HCC-4	C-C motif chemokine 16	Chemokine	1.09 (0.95 to 1.24)	0.2192
4922_13	CCL19	IVW	26	MIP-3b	C-C motif chemokine 19	Chemokine	1.03 (0.78 to 1.35)	0.8466
5301_7	CCL11	IVW	28	Eotaxin	Eotaxin	Chemokine	1.22 (0.91 to 1.64)	0.1742
5480_49	CCL5	IVW	13	RANTES	C-C motif chemokine 5	Chemokine	1.03 (0.66 to 1.63)	0.8855

5730_60	CXCL14	Wald ratio	1	BRAK	C-X-C motif chemokine 14	Chemokine	1.24 (0.17 to 8.87)	0.8273
9168_31	CCL26	IVW	7	Eotaxin-3	C-C motif chemokine 26	Chemokine	1.06 (0.52 to 2.17)	0.8743
9495_10	CXCL17	IVW	8	VCC1	C-X-C motif chemokine 17	Chemokine	1.04 (0.63 to 1.73)	0.8779
11219_95	FGFBP3	IVW	18	FGFP3	Fibroblast growth factor-binding protein 3	Growth factor	0.82 (0.57 to 1.17)	0.2678
13098_93	FIGF	IVW	6	VEGF-D	Vascular endothelial growth factor D	Growth factor	1.89 (0.86 to 4.18)	0.1156
13669_6	FGFR3	IVW	11	FGFR-3	Fibroblast growth factor receptor 3	Growth factor	0.81 (0.49 to 1.32)	0.3886
13724_27	FGF19	IVW	11	FGF-19	Fibroblast growth factor 19	Growth factor	1.00 (0.68 to 1.49)	0.9874
15494_11	FGFBP1	IVW	9	FGFP1	Fibroblast growth factor-binding protein 1	Growth factor	1.12 (0.53 to 2.40)	0.7644
17166_4	FGF8	IVW	2	FGF-8F	Fibroblast growth factor 8 isoform F	Growth factor	0.45 (0.04 to 4.74)	0.5102
2597_8	VEGFA	IVW	51	VEGF	Vascular endothelial growth factor A	Growth factor	0.94 (0.78 to 1.13)	0.5032
2681_23	HGF	IVW	6	HGF	Hepatocyte growth factor	Growth factor	0.74 (0.37 to 1.47)	0.3873
2761_49	FGF18	Wald ratio	1	FGF-18	Fibroblast growth factor 18	Growth factor	1.26 (0.14 to 10.94)	0.8362
2763_66	FGF20	IVW	3	FGF-20	Fibroblast growth factor 20	Growth factor	1.08 (0.21 to 5.41)	0.9285
2966_65	CLEC11A	IVW	40	SCGF-beta	Stem cell growth factor-beta	Growth factor	0.99 (0.74 to 1.30)	0.9158
3025_50	FGF2	IVW	54	bFGF	Fibroblast growth factor 2	Growth factor	1.06 (0.87 to 1.30)	0.5474
3065_65	FGF5	IVW	4	FGF-5	Fibroblast growth factor 5	Growth factor	0.93 (0.41 to 2.13)	0.8619
3132_1	VEGFC	Wald ratio	1	VEGF-C	Vascular endothelial growth factor C	Growth factor	1.04 (0.53 to 2.04)	0.9006
3486_58	FGF1	IVW	4	b-ECGF	Fibroblast growth factor 1	Growth factor	1.12 (0.41 to 3.04)	0.8209
3494_71	FGF17	IVW	6	FGF-17	Fibroblast growth factor 17	Growth factor	1.21 (0.60 to 2.43)	0.5990
3617_80	HGFAC	IVW	66	HGFA	Hepatocyte growth factor activator	Growth factor	1.00 (0.87 to 1.15)	0.9876
3651_50	KDR	IVW	42	VEGF sR2	Vascular endothelial growth factor receptor 2	Growth factor	0.89 (0.74 to 1.08)	0.2285
3807_1	FGF23	IVW	2	FGF23	Fibroblast growth factor 23	Growth factor	3.81 (0.51 to 28.54)	0.1929
3808_76	FGFR2	IVW	2	FGFR-2	Fibroblast growth factor receptor 2	Growth factor	0.25 (0.04 to 1.59)	0.1411
4123_60	FGF4	Wald ratio	1	FGF-4	Fibroblast growth factor 4	Growth factor	0.13 (0.00 to 5.83)	0.2891
4392_54	FGF12	IVW	2	FGF-12	Fibroblast growth factor 12	Growth factor	0.82 (0.15 to 4.42)	0.8133
4393_3	FGF16	IVW	2	FGF-16	Fibroblast growth factor 16	Growth factor	1.24 (0.22 to 7.07)	0.8097
4487_1	FGF7	IVW	3	FGF7	Fibroblast growth factor 7	Growth factor	0.55 (0.09 to 3.40)	0.5175
4988_49	FGFR4	IVW	6	FGFR4	Fibroblast growth factor receptor 4	Growth factor	0.70 (0.30 to 1.59)	0.3904
5532_53	FGFR1	IVW	19	bFGF-R	Fibroblast growth factor receptor 1	Growth factor	1.31 (0.82 to 2.08)	0.2529

5801_72	NGF	Wald ratio	1	b-NGF	beta-nerve growth factor	Growth factor	0.91 (0.12 to 7.04)	0.9286
6237_11	FGFRL1	IVW	4	FGRL1	Fibroblast growth factor receptor-like 1	Growth factor	0.61 (0.20 to 1.83)	0.3807
7894_155	FGF3	IVW	3	FGF-3	Fibroblast growth factor 3	Growth factor	1.15 (0.43 to 3.06)	0.7783
16315_105	FLT1	IVW	27	VEGF sR1	Vascular endothelial growth factor receptor 1	Growth factor	1.06 (0.80 to 1.40)	0.6848
9453_12	VEGFB	Wald ratio	1	VEGF-B	Vascular endothelial growth factor B	Growth factor	1.07 (0.16 to 7.32)	0.9445
14127_240	IFNB1	IVW	2	IFN-b	Interferon beta	Interferon	1.42 (0.29 to 6.87)	0.6645
14128_121	IFNA10	IVW	3	IFN10	Interferon alpha-10	Interferon	1.25 (0.42 to 3.70)	0.6812
14129_1	IFNA7	Wald ratio	1	IFNA7	Interferon alpha-7	Interferon	0.22 (0.01 to 4.11)	0.3083
15346_31	IFNG	IVW	5	IFN-g	Interferon gamma	Interferon	1.18 (0.61 to 2.25)	0.6264
15405_23	IFNA4	IVW	3	IFNA4	Interferon alpha-4	Interferon	1.18 (0.39 to 3.57)	0.7704
18389_11	IFNA1	IVW	3	IFNA1	Interferon alpha-1/13	Interferon	1.40 (0.46 to 4.28)	0.5505
3497_13	IFNA2	IVW	8	IFN-aA	Interferon alpha-2	Interferon	1.05 (0.65 to 1.69)	0.8413
4396_54	IFNL1	IVW	2	IFN-lambda 1	Interferon lambda-1	Interferon	1.24 (0.22 to 7.13)	0.8109
4397_26	IFNL2	IVW	2	IFN-lambda 2	Interferon lambda-2	Interferon	1.18 (0.27 to 5.13)	0.8248
5713_9	IFNL3	IVW	5	IFN-lambda 3	Interferon lambda-3	Interferon	1.92 (0.70 to 5.23)	0.2035
5714_88	IFNA6	IVW	4	IFNA6	Interferon alpha-6	Interferon	1.07 (0.44 to 2.59)	0.8813
5825_49	IFNGR1	IVW	5	IFN-g R1	Interferon gamma receptor 1	Interferon	1.23 (0.39 to 3.89)	0.7200
6210_100	IFNA5	IVW	11	IFNA5	Interferon alpha-5	Interferon	0.89 (0.55 to 1.44)	0.6356
7180_114	IFNA14	IVW	2	IFN14	Interferon alpha-14	Interferon	1.38 (0.35 to 5.47)	0.6467
7192_37	IFNLR1	IVW	6	CRF2-12	Interferon lambda receptor 1	Interferon	0.70 (0.38 to 1.29)	0.2514
8818_13	IFNGR2	Wald ratio	1	INGR2	Interferon gamma receptor 2	Interferon	0.25 (0.02 to 3.66)	0.3083
9183_7	IFNAR1	IVW	50	IFN-a/b R1	Interferon alpha/beta receptor 1	Interferon	0.89 (0.75 to 1.04)	0.1437
10344_334	IL10RA	IVW	23	IL-10 Ra	Interleukin-10 receptor subunit alpha	Interleukin	0.85 (0.63 to 1.13)	0.2579
10455_196	IL31	IVW	22	IL-31	Interleukin-31	Interleukin	0.77 (0.54 to 1.11)	0.1659
11071_1	IL5	IVW	3	IL-5	Interleukin-5	Interleukin	1.20 (0.28 to 5.13)	0.8077
12665_16	ILF2	Wald ratio	1	ILF2	Interleukin enhancer-binding factor 2	Interleukin	1.27 (0.13 to 12.44)	0.8362
12759_47	ILF3	IVW	3	DRBP76	Interleukin enhancer-binding factor 3	Interleukin	1.15 (0.37 to 3.56)	0.8084
13435_31	IL20RB	IVW	12	IL-20 Rb	Interleukin-20 receptor subunit beta	Interleukin	1.12 (0.64 to 1.95)	0.6926
13686_2	IL5RA	IVW	57	IL-5 Ra	Interleukin-5 receptor subunit alpha	Interleukin	0.83 (0.68 to 1.01)	0.0626

13733_5	IL12B	IVW	52	IL-12 p40	Interleukin-12 subunit beta	Interleukin	0.96 (0.78 to 1.17)	0.6549
13744_37	IL3RA	IVW	72	IL-3 Ra	Interleukin-3 receptor subunit alpha	Interleukin	1.05 (0.90 to 1.23)	0.5538
14026_24	IL17F	IVW	5	IL-17F	Interleukin-17F	Interleukin	1.04 (0.45 to 2.43)	0.9242
14048_7	IL1RAP	IVW	69	IL-1 R AcP	Interleukin-1 Receptor accessory protein	Interleukin	1.07 (0.95 to 1.19)	0.2550
14054_17	IL15RA	IVW	42	IL-15 Ra	Interleukin-15 receptor subunit alpha	Interleukin	1.13 (0.91 to 1.40)	0.2581
14079_14	IL18R1	IVW	66	IL-18 Ra	Interleukin-18 receptor 1	Interleukin	0.93 (0.78 to 1.12)	0.4621
14133_93	IL1R2	IVW	51	IL-1 sRII	Interleukin-1 receptor type 2	Interleukin	0.89 (0.70 to 1.12)	0.3146
14149_9	IL36B	IVW	4	IL-1F8	Interleukin-36 beta	Interleukin	1.37 (0.60 to 3.11)	0.4504
14150_7	IL36A	IVW	22	IL-1F6	Interleukin-36 alpha	Interleukin	1.04 (0.75 to 1.45)	0.7948
15602_43	IL6R	IVW	99	IL-6 sRa	Interleukin-6 receptor subunit alpha	Interleukin	0.89 (0.79 to 1.00)	0.0517
17356_34	IL1F10	IVW	5	IL1FA	Interleukin-1 family member 10	Interleukin	1.13 (0.41 to 3.11)	0.8162
18216_22	IL11RA	IVW	35	IL-11 RA	Interleukin-11 receptor subunit alpha	Interleukin	0.90 (0.69 to 1.19)	0.4629
18375_28	IL1F5	IVW	29	IL-1F5	Interleukin-36 receptor antagonist protein	Interleukin	1.00 (0.74 to 1.35)	0.9973
19568_17	IL15	IVW	9	IL-15	Interleukin-15	Interleukin	0.93 (0.52 to 1.65)	0.7980
2631_50	IL10RB	IVW	12	IL-10 Rb	Interleukin-10 receptor subunit beta	Interleukin	0.91 (0.59 to 1.39)	0.6534
2632_5	IL12RB1	IVW	7	IL-12 Rb1	Interleukin-12 receptor subunit beta-1	Interleukin	1.08 (0.58 to 2.02)	0.8048
2633_52	IL13RA1	IVW	2	IL-13 Ra1	Interleukin-13 receptor subunit alpha-1	Interleukin	0.99 (0.14 to 6.80)	0.9896
2773_50	IL10	IVW	2	IL-10	Interleukin-10	Interleukin	0.96 (0.18 to 5.17)	0.9614
2778_10	IL22	Wald ratio	1	IL-22	Interleukin-22	Interleukin	0.45 (0.03 to 7.04)	0.5668
2906_55	IL4	IVW	3	IL-4	Interleukin-4	Interleukin	1.25 (0.38 to 4.16)	0.7168
2991_9	IL1R1	IVW	34	IL-1 sRI	Interleukin-1 receptor type 1	Interleukin	0.99 (0.74 to 1.33)	0.9674
2992_59	IL17RA	IVW	63	IL-17 sR	Interleukin-17 receptor A	Interleukin	1.03 (0.92 to 1.16)	0.5906
2993_1	IL18RAP	IVW	5	IL-18 Rb	Interleukin-18 receptor accessory protein	Interleukin	1.39 (0.66 to 2.94)	0.3838
2994_71	IL1RL2	IVW	6	IL-1Rrp2	Interleukin-1 receptor-like 2	Interleukin	0.83 (0.45 to 1.53)	0.5524
3035_80	IL19	IVW	48	IL-19	Interleukin-19	Interleukin	0.99 (0.86 to 1.13)	0.8309
3037_62	IL1B	IVW	13	IL-1b	Interleukin-1 beta	Interleukin	1.03 (0.77 to 1.37)	0.8505
3070_1	IL2	IVW	5	IL-2	Interleukin-2	Interleukin	0.46 (0.16 to 1.33)	0.1507
3072_4	IL13	IVW	5	IL-13	Interleukin-13	Interleukin	1.06 (0.50 to 2.24)	0.8728
3073_51	IL18BP	IVW	10	IL-18 BPa	Interleukin-18-binding protein	Interleukin	1.15 (0.65 to 2.04)	0.6312

3151_6	IL2RA	IVW	2	IL-2 sRa	Interleukin-2 receptor subunit alpha	Interleukin	1.27 (0.25 to 6.55)	0.7754
3321_2	IL24	Wald ratio	1	IL24	Interleukin-24	Interleukin	1.24 (0.16 to 9.83)	0.8378
3376_49	IL17RD	IVW	32	IL-17 RD	Interleukin-17 receptor D	Interleukin	1.07 (0.84 to 1.36)	0.6017
3447_64	CXCL8	IVW	7	IL-8	Interleukin-8	Interleukin	0.85 (0.40 to 1.80)	0.6783
3499_77	IL17B	IVW	6	IL-17B	Interleukin-17B	Interleukin	1.26 (0.55 to 2.92)	0.5860
3620_67	IL22RA1	IVW	7	IL22RA1	Interleukin-22 receptor subunit alpha-1	Interleukin	1.14 (0.62 to 2.09)	0.6658
3815_14	IL12RB2	Wald ratio	1	IL-12 RB2	Interleukin-12 receptor subunit beta-2	Interleukin	0.09 (0.00 to 2.62)	0.1604
4136_40	IL17D	IVW	2	IL-17D	Interleukin-17D	Interleukin	1.20 (0.35 to 4.13)	0.7751
4137_57	IL25	IVW	5	IL-17E	Interleukin-25	Interleukin	0.82 (0.49 to 1.37)	0.4420
4138_25	IL20	Wald ratio	1	IL-20	Interleukin-20	Interleukin	1.21 (0.20 to 7.14)	0.8362
4140_3	IL7	IVW	3	IL-7	Interleukin-7	Interleukin	1.12 (0.29 to 4.33)	0.8674
4234_8	IL1RL1	IVW	125	IL-1 R4	Interleukin-1 receptor-like 1	Interleukin	0.97 (0.85 to 1.10)	0.6414
4493_92	IL11	Wald ratio	1	IL-11	Interleukin-11	Interleukin	1.08 (0.28 to 4.09)	0.9131
4556_10	IL34	IVW	25	IL-34	Interleukin-34	Interleukin	0.92 (0.66 to 1.28)	0.6176
4673_13	IL6	Wald ratio	1	IL-6	Interleukin-6	Interleukin	0.46 (0.05 to 4.65)	0.5128
4717_55	IL3	IVW	3	IL-3	Interleukin-3	Interleukin	0.88 (0.22 to 3.44)	0.8489
4851_25	IL1A	Wald ratio	1	IL-1a	Interleukin-1 alpha	Interleukin	0.79 (0.07 to 9.44)	0.8513
5082_51	IL1RAPL2	IVW	3	IL-1 sR9	X-linked interleukin-1 receptor accessory protein-like 2	Interleukin	1.29 (0.40 to 4.14)	0.6655
5085_18	IL20RA	Wald ratio	1	IL-20 Ra	Interleukin-20 receptor subunit alpha	Interleukin	1.46 (0.14 to 15.65)	0.7567
5087_5	IL22RA2	IVW	49	IL-22BP	Interleukin-22 receptor subunit alpha-2	Interleukin	0.97 (0.79 to 1.20)	0.7819
**5088_175**	**IL23R**	**IVW**	**10**	**IL-23 R**	**Interleukin-23 receptor**	**Interleukin**	**0.60 (0.38 to 0.95)**	**0.0282**
5089_11	IL7R	IVW	6	IL-7 Ra	Interleukin-7 receptor subunit alpha	Interleukin	1.21 (0.53 to 2.75)	0.6453
**5132_71**	**IL27RA**	**IVW**	**55**	**TCCR**	**Interleukin-27 receptor subunit alpha**	**Interleukin**	**1.16 (1.01 to 1.33)**	**0.0359**
5353_89	IL1RN	IVW	8	IL-1Ra	Interleukin-1 receptor antagonist protein	Interleukin	1.06 (0.63 to 1.78)	0.8254
5468_67	IL17RC	IVW	3	IL-17 RC	Interleukin-17 receptor C	Interleukin	1.27 (0.27 to 5.98)	0.7636
5661_15	IL18	Wald ratio	1	IL-18	Interleukin-18	Interleukin	1.65 (0.12 to 21.83)	0.7052
5834_18	IL9	IVW	5	IL-9	Interleukin-9	Interleukin	0.95 (0.27 to 3.38)	0.9413
6262_14	IL17RB	IVW	5	IL-17B R	Interleukin-17 receptor B	Interleukin	0.52 (0.21 to 1.30)	0.1615
7124_18	IL21	IVW	18	IL-21	Interleukin-21	Interleukin	1.06 (0.76 to 1.48)	0.7155

8273_84	IL31RA	IVW	4	IL31R	Interleukin-31 receptor subunit alpha	Interleukin	1.21 (0.39 to 3.71)	0.7397
9051_13	IL32	Wald ratio	1	IL32	Interleukin-32	Interleukin	1.12 (0.39 to 3.16)	0.8362
9117_4	IL36G	Wald ratio	1	IL-1F9	Interleukin-36 gamma	Interleukin	1.26 (0.14 to 11.08)	0.8362
9170_24	IL17A	IVW	2	IL-17A	Interleukin-17A	Interleukin	0.64 (0.12 to 3.48)	0.6021
9255_5	IL17C	IVW	5	IL-17C	Interleukin-17C	Interleukin	1.29 (0.62 to 2.67)	0.4943
9343_16	IL2RB	IVW	16	IL-2 sRb	Interleukin-2 receptor subunit beta	Interleukin	1.12 (0.75 to 1.67)	0.5895
9366_54	IL21R	IVW	2	IL-21 sR	Interleukin-21 receptor	Interleukin	1.00 (0.30 to 3.26)	0.9958
13682_47	CSF1R	IVW	38	M-CSF R	Macrophage colony-stimulating factor 1 receptor	Others	0.97 (0.67 to 1.39)	0.8634
2719_3	CSF3R	IVW	8	G-CSF-R	Granulocyte colony-stimulating factor receptor	Others	1.08 (0.65 to 1.78)	0.7757
2925_9	SERPINE1	IVW	9	PAI-1	Plasminogen activator inhibitor 1	Others	0.51 (0.26 to 1.01)	0.0520
3738_54	CSF1	IVW	4	CSF-1	Macrophage colony-stimulating factor 1	Others	1.05 (0.50 to 2.19)	0.9031
4840_73	CSF3	IVW	35	G-CSF	Granulocyte colony-stimulating factor	Others	0.99 (0.78 to 1.27)	0.9634
8221_19	MIF	IVW	2	MIF	Macrophage migration inhibitory factor	Others	0.42 (0.09 to 1.92)	0.2601
11837_7	TNFRSF18	IVW	2	GITR	Tumor necrosis factor receptor superfamily member 18	Tumor necrosis factor	2.66 (0.42 to 17.01)	0.3005
12563_2	TNFAIP8	IVW	4	TFIP8	Tumor necrosis factor alpha-induced protein 8	Tumor necrosis factor	1.40 (0.72 to 2.72)	0.3190
14009_65	TNFAIP3	IVW	5	TNFAIP3	Tumor necrosis factor alpha-induced protein 3	Tumor necrosis factor	0.70 (0.19 to 2.61)	0.5961
14025_18	TNFRSF9	IVW	3	4-1BB	Tumor necrosis factor receptor superfamily member 9	Tumor necrosis factor	1.49 (0.47 to 4.77)	0.4971
14061_48	TNFSF11	IVW	7	sRANKL	Tumor necrosis factor ligand superfamily member 11	Tumor necrosis factor	1.00 (0.53 to 1.90)	0.9998
14121_24	TNFRSF10D	IVW	8	TRAIL R4	Tumor necrosis factor receptor superfamily member 10D	Tumor necrosis factor	0.94 (0.51 to 1.74)	0.8542
2654_19	TNFRSF1A	IVW	11	TNF sR-I	Tumor necrosis factor receptor superfamily member 1A	Tumor necrosis factor	1.16 (0.61 to 2.19)	0.6485
2665_26	TNFRSF17	IVW	21	BCMA	Tumor necrosis factor receptor superfamily member 17	Tumor necrosis factor	1.38 (0.90 to 2.12)	0.1348
2704_74	TNFRSF13B	IVW	7	TACI	Tumor necrosis factor receptor superfamily member 13B	Tumor necrosis factor	1.13 (0.65 to 1.97)	0.6635
2708_54	TNFSF18	IVW	10	TNFSF18	Tumor necrosis factor ligand superfamily member 18	Tumor necrosis factor	1.03 (0.67 to 1.58)	0.8980
2839_2	TNFSF4	IVW	3	OX40 Ligand	Tumor necrosis factor ligand superfamily member 4	Tumor necrosis factor	1.40 (0.43 to 4.55)	0.5785
2968_61	TNFSF15	IVW	2	TNFSF15	Tumor necrosis factor ligand superfamily member 15	Tumor necrosis factor	4.04 (0.47 to 34.48)	0.2013
3059_50	TNFSF13B	IVW	10	BAFF	Tumor necrosis factor ligand superfamily member 13B	Tumor necrosis factor	1.24 (0.61 to 2.49)	0.5517
3152_57	TNFRSF1B	IVW	10	TNF sR-II	Tumor necrosis factor receptor superfamily member 1B	Tumor necrosis factor	1.08 (0.56 to 2.10)	0.8141
3421_54	TNFSF8	IVW	21	CD30 Ligand	Tumor necrosis factor ligand superfamily member 8	Tumor necrosis factor	0.98 (0.67 to 1.42)	0.9053
3730_81	TNFRSF4	IVW	2	TNR4	Tumor necrosis factor receptor superfamily member 4	Tumor necrosis factor	1.12 (0.20 to 6.14)	0.8967

4703_87	LTA	IVW	3	TNF-b	Lymphotoxin-alpha	Tumor necrosis factor	1.24 (0.39 to 3.88)	0.7158
4832_75	TNFRSF10A	IVW	2	TRAIL R1	Tumor necrosis factor receptor superfamily member 10A	Tumor necrosis factor	0.56 (0.06 to 4.88)	0.5991
5036_50	TNFAIP6	IVW	78	TSG-6	Tumor necrosis factor-inducible gene 6 protein	Tumor necrosis factor	1.02 (0.87 to 1.20)	0.7640
5070_76	TNFRSF6B	IVW	3	DcR3	Tumor necrosis factor receptor superfamily member 6B	Tumor necrosis factor	0.87 (0.22 to 3.39)	0.8359
5131_15	TNFRSF19	IVW	4	TAJ	Tumor necrosis factor receptor superfamily member 19	Tumor necrosis factor	0.50 (0.23 to 1.09)	0.0825
5138_50	TNFRSF12A	Wald ratio	1	TWEAKR	Tumor necrosis factor receptor superfamily member 12A	Tumor necrosis factor	0.25 (0.02 to 3.57)	0.3083
5352_11	TNFRSF14	IVW	2	HVEM	Tumor necrosis factor receptor superfamily member 14	Tumor necrosis factor	1.29 (0.41 to 4.00)	0.6634
5355_69	TNFSF14	IVW	6	LIGHT	Tumor necrosis factor ligand superfamily member 14	Tumor necrosis factor	0.90 (0.41 to 1.96)	0.7907
5383_14	TNFRSF13C	IVW	4	BAFF Receptor	Tumor necrosis factor receptor superfamily member 13C	Tumor necrosis factor	0.82 (0.44 to 1.54)	0.5434
5404_53	TNFRSF21	IVW	17	DR6	Tumor necrosis factor receptor superfamily member 21	Tumor necrosis factor	0.81 (0.49 to 1.32)	0.3906
5534_49	TNFRSF10B	IVW	7	TRAIL R2	Tumor necrosis factor receptor superfamily member 10B	Tumor necrosis factor	0.89 (0.49 to 1.62)	0.7099
5936_53	TNF	IVW	3	TNF-a	Tumor necrosis factor	Tumor necrosis factor	1.11 (0.26 to 4.73)	0.8889
5939_42	TNFSF12	IVW	18	TWEAK	Tumor necrosis factor ligand superfamily member 12	Tumor necrosis factor	1.01 (0.71 to 1.44)	0.9639
8304_50	TNFRSF11B	IVW	26	OPG	Tumor necrosis factor receptor superfamily member 11B	Tumor necrosis factor	1.13 (0.67 to 1.89)	0.6539
8833_20	TNFSF10	IVW	5	TRAIL	Tumor necrosis factor ligand superfamily member 10	Tumor necrosis factor	1.08 (0.45 to 2.60)	0.8602

* protein ID in the Nat Genet. 2021;53(12):1712-21

Table S4. Associations of inflammatory traits with liver cancer (UKBB) according to Mendelian randomization analysis.

Inflammatory trait	method	No. of IVs	Biomarker	Full name	Group	lower	OR	upper	P value
**C-reactive protein**	IVW	287			Circulating inflammatory biomarkers	0.87	1.14	1.61	0.155
**Neutrophil count**	IVW	161			Circulating inflammatory biomarkers	0.78	1.25	1.93	0.149
**Leukocyte count**	IVW	183			Circulating inflammatory biomarkers	0.52	1.02	1.28	0.167
**Monocyte count**	IVW	259			Circulating inflammatory biomarkers	0.53	0.98	1.42	0.370
**Eosinophil count**	IVW	204			Circulating inflammatory biomarkers	0.51	1.15	1.61	0.376
**Lymphocyte count**	IVW	185			Circulating inflammatory biomarkers	0.62	0.94	1.29	0.788
**Basophil count**	IVW	80			Circulating inflammatory biomarkers	0.79	0.95	1.23	0.795
**Type 1 diabetes**	IVW	130			Immune-mediated diseases	0.67	1.04	1.11	0.106
**Multiple sclerosis**	IVW	53			Immune-mediated diseases	0.51	1.05	1.62	0.252
**Rheumatoid arthritis**	IVW	117			Immune-mediated diseases	0.64	1.04	1.43	0.321
**Asthma**	IVW	224			Immune-mediated diseases	0.61	1.07	1.34	0.456
**Crohn's disease**	IVW	106			Immune-mediated diseases	0.82	1.29	1.91	0.457
**Ulcerative colitis**	IVW	76			Immune-mediated diseases	0.83	1.02	1.52	0.525
**Psoriasis**	IVW	84			Immune-mediated diseases	0.68	1.17	1.41	0.560
**Celiac disease**	IVW	11			Immune-mediated diseases	0.56	0.89	1.25	0.778
**Systemic lupus erythematosus**	IVW	47			Immune-mediated diseases	0.44	1.02	1.76	0.254
**CXCL17**	IVW	8	VCC1	C-X-C motif chemokine 17	Chemokine	**1.06**	**1.45**	**1.79**	**0.011**
**CCL25**	IVW	56	TECK	C-C motif chemokine 25	Chemokine	0.46	1.06	1.52	0.099
**CCL17**	IVW	44	TARC	C-C motif chemokine 17	Chemokine	0.52	1.16	1.38	0.525
**CCL5**	IVW	13	RANTES	C-C motif chemokine 5	Chemokine	0.91	1.21	1.83	0.168
**CCL18**	IVW	48	PARC	C-C motif chemokine 18	Chemokine	0.96	1.49	1.71	0.447
**CCL23**	IVW	25	MPIF-1	C-C motif chemokine 23	Chemokine	0.47	1.11	1.15	0.427
**CCL15**	IVW	54	MIP-5	C-C motif chemokine 15	Chemokine	0.35	1.15	1.33	0.686
**CCL19**	IVW	26	MIP-3b	C-C motif chemokine 19	Chemokine	0.27	0.84	1.68	0.058
**CCL3**	IVW	11	MIP-1a	C-C motif chemokine 3	Chemokine	0.66	0.95	1.08	0.761
**CXCL9**	IVW	4	MIG	C-X-C motif chemokine 9	Chemokine	0.26	1.24	1.90	0.210

**CCL22**	IVW	33	MDC	C-C motif chemokine 22	Chemokine	0.53	0.85	1.81	0.945
**CCL13**	IVW	2	MCP-4	C-C motif chemokine 13	Chemokine	0.53	0.91	1.22	0.519
**CCL7**	IVW	42	MCP-3	C-C motif chemokine 7	Chemokine	0.43	0.90	1.05	0.261
**CCL8**	IVW	81	MCP-2	C-C motif chemokine 8	Chemokine	0.45	0.97	1.74	0.983
**CCL2**	IVW	4	MCP-1	C-C motif chemokine 2	Chemokine	0.88	1.03	1.45	0.171
**CCL4L1**	IVW	11	LAG-1	C-C motif chemokine 4-like	Chemokine	0.79	1.12	1.27	0.589
**CXCL11**	IVW	36	I-TAC	C-X-C motif chemokine 11	Chemokine	0.85	1.33	1.72	0.993
**CXCL10**	IVW	11	IP-10	C-X-C motif chemokine 10	Chemokine	0.83	1.14	1.60	0.436
**CCL1**	IVW	22	I-309	C-C motif chemokine 1	Chemokine	0.62	1.10	1.27	0.523
**CCL16**	IVW	64	HCC-4	C-C motif chemokine 16	Chemokine	0.47	0.98	1.35	0.236
**CCL14**	IVW	30	HCC-1	C-C motif chemokine 14	Chemokine	0.71	1.36	1.50	0.234
**CXCL6**	IVW	70	GCP-2	C-X-C motif chemokine 6	Chemokine	0.46	1.24	1.48	0.989
**CCL26**	IVW	7	Eotaxin-3	C-C motif chemokine 26	Chemokine	0.41	1.24	1.65	0.101
**CCL11**	IVW	28	Eotaxin	Eotaxin	Chemokine	0.27	1.28	1.84	0.165
**CXCL5**	IVW	8	ENA-78	C-X-C motif chemokine 5	Chemokine	0.26	1.12	1.40	0.745
**CXCL16**	IVW	41	CXCL16, soluble	C-X-C motif chemokine 16	Chemokine	0.44	0.88	1.12	0.621
**CCL27**	IVW	2	CTACK	C-C motif chemokine 27	Chemokine	0.24	1.15	1.93	0.573
**CXCL14**	Wald ratio	1	BRAK	C-X-C motif chemokine 14	Chemokine	0.41	1.40	1.96	0.906
**CXCL13**	IVW	4	BLC	C-X-C motif chemokine 13	Chemokine	0.58	0.54	1.53	0.310
**CCL21**	IVW	56	6Ckine	C-C motif chemokine 21	Chemokine	0.24	0.88	1.98	0.893
**FIGF**	IVW	6	VEGF-D	Vascular endothelial growth factor D	Growth factor	0.83	1.11	1.27	0.684
**VEGFC**	Wald ratio	1	VEGF-C	Vascular endothelial growth factor C	Growth factor	0.57	1.25	1.82	0.031
**VEGFB**	Wald ratio	1	VEGF-B	Vascular endothelial growth factor B	Growth factor	0.76	1.11	1.39	0.069
**KDR**	IVW	42	VEGF sR2	Vascular endothelial growth factor receptor 2	Growth factor	0.75	1.23	1.93	0.559
**FLT1**	IVW	27	VEGF sR1	Vascular endothelial growth factor receptor 1	Growth factor	0.45	1.24	1.79	0.388
**VEGFA**	IVW	51	VEGF	Vascular endothelial growth factor A	Growth factor	0.88	1.16	1.99	0.499
**CLEC11A**	IVW	40	SCGF-beta	Stem cell growth factor-beta	Growth factor	0.58	0.88	1.37	0.375
**HGFAC**	IVW	66	HGFA	Hepatocyte growth factor activator	Growth factor	0.92	1.38	1.95	0.621
**HGF**	IVW	6	HGF	Hepatocyte growth factor	Growth factor	0.94	1.25	1.27	0.308

**FGFRL1**	IVW	4	FGRL1	Fibroblast growth factor receptor-like 1	Growth factor	0.42	1.14	1.80	0.664
**FGFR4**	IVW	6	FGFR4	Fibroblast growth factor receptor 4	Growth factor	0.28	0.98	1.48	0.331
**FGFR3**	**IVW**	**11**	**FGFR-3**	**Fibroblast growth factor receptor 3**	**Growth factor**	**1.03**	**1.23**	**1.41**	**0.039**
**FGFR2**	IVW	2	FGFR-2	Fibroblast growth factor receptor 2	Growth factor	0.79	0.95	1.14	0.270
**FGFBP3**	IVW	18	FGFP3	Fibroblast growth factor-binding protein 3	Growth factor	0.61	0.98	1.29	0.801
**FGFBP1**	IVW	9	FGFP1	Fibroblast growth factor-binding protein 1	Growth factor	0.78	1.08	1.31	0.801
**FGF8**	IVW	2	FGF-8F	Fibroblast growth factor 8 isoform F	Growth factor	0.56	1.14	1.28	0.797
**FGF7**	IVW	3	FGF7	Fibroblast growth factor 7	Growth factor	0.85	1.05	1.30	0.829
**FGF5**	IVW	4	FGF-5	Fibroblast growth factor 5	Growth factor	0.75	1.01	1.34	0.204
**FGF4**	Wald ratio	1	FGF-4	Fibroblast growth factor 4	Growth factor	0.35	1.00	1.14	0.666
**FGF3**	IVW	3	FGF-3	Fibroblast growth factor 3	Growth factor	0.90	1.11	1.83	0.821
**FGF23**	IVW	2	FGF23	Fibroblast growth factor 23	Growth factor	0.87	1.42	1.97	0.765
**FGF20**	IVW	3	FGF-20	Fibroblast growth factor 20	Growth factor	0.70	1.09	1.67	0.727
**FGF19**	IVW	11	FGF-19	Fibroblast growth factor 19	Growth factor	0.60	1.14	1.33	0.458
**FGF18**	Wald ratio	1	FGF-18	Fibroblast growth factor 18	Growth factor	0.57	1.03	1.40	0.423
**FGF17**	IVW	6	FGF-17	Fibroblast growth factor 17					
